# Selection criteria and match results for postgraduate residency programs: A cross-sectional model from a major academic center in Jordan

**DOI:** 10.1016/j.amsu.2020.10.001

**Published:** 2020-10-07

**Authors:** Wasim Khasawneh, Nail Obeidat, Borhan Albiss, Khalid El-Salem

**Affiliations:** a Department of Pediatrics and Neonatology, Faculty of Medicine, Jordan University of Science and Technology, Irbid, Jordan; b Department of Obstetrics and Gynecology, Faculty of Medicine, Jordan University of Science and Technology, Irbid, Jordan; c Department of Applied Physical Sciences, Faculty of Graduate Studies, Jordan University of Science and Technology, Irbid, Jordan; d Department of Neurosciences, Faculty of Medicine, Jordan University of Science and Technology, Irbid, Jordan

**Keywords:** Selection criteria, Residency programs, Jordan, Match results

## Abstract

**Background:**

With the continuous uptrend in the number of medical student graduates and the limited availability of postgraduate residency positions, the process of selecting the most appropriately qualified candidates to fill these positions remains challenging. This necessitates implementing objectively measured, distinguishing, and transparent selection process. The purpose of this study is to share our model of single-center resident selection for postgraduate residency programs to serve as a guide for other institutions.

**Materials and methods:**

We reviewed the process of residency program selection at our institution. Data were collected about the application process, demographic characteristics, medical school location and GPA, entry exam score, requested specialties, and match results. The proposed selection criteria and their association with the match results were reported. Factors associated with matching with the first two selections were analyzed.

**Results:**

785 physicians applied to fill 96 positions at nineteen residency programs. 443 (56%) were males, 686 (87%) graduated from Jordanian medical schools. Half failed the entry exam and were excluded from competition. Seventy-two out of 96 (75%) matched with either of their first two requested specialties. The highest-in-demand programs were ophthalmology, otolaryngology and dermatology. Although a GPA of more than 80% increased the likelihood of matching with the top two requested specialties, an entry exam score of more than 70% was the main determining factor (AOR 8.7, 95% C.I. 2.4, 31.9).

**Conclusions:**

The selection process for postgraduate residency programs is highly competitive. To avoid selection bias, transparent and objectively measured criteria are applied in the selection model. Clinical performance and medical knowledge reflected by the cumulative GPA and entry exam score are the most significant determinants for acceptance.

## Highlights

• There is a global uptrend in the number of medical student graduates.
• Selecting the most qualified candidates to fill postgraduate positions remains challenging.
• Globally, there are no uniformly accepted criteria for selection of the most suitable candidates.
• Implementation of objectively measured criteria helps to minimize selection bias.

## 1 Introduction

Postgraduate residency programs are conducted all across the world in order to graduate competent and highly skilled physicians capable of providing the best healthcare services and to participate in academic jobs by teaching medical students and conducting scientific research and publication production.

In Jordan, acceptance into a medical school is mainly determined by the score achieved during the last year of high school named as Tawjihi [1]. The number of students who joined the medical schools in Jordan is up-trending. Currently, there are six medical schools in the country which accommodated nearly four thousand medical students in the year 2019–2020 [2], [3]. According to the most recent statistics, the number of medical students who graduated from the Jordanian medical schools in 2019 approximated 1600. Additionally, more than one thousand Jordanian students graduated from non-Jordanian medical schools and came back to practice medicine in Jordan [2].

After graduation from the medical school, the doctor of Medicine (MD) bachelor degree holders are required to complete a one-year internship in order to obtain unrestricted license to practice medicine in the country. Thereafter, licensees have the option of practicing general medicine through the general practitioner track or joining a residency program that offers postgraduate training. In Jordan, postgraduate residency programs are offered through three academic institutions, Ministry of health hospitals, Military hospitals via Royal Medical services, or through certain hospitals in the private health sector [3]. All hospitals and institutions are registered through the Jordanian Medical Council (JMC) and must be compliant with all the requirements set by the council to be approved to host residency training programs [4]. Among the Jordanian academic institutions is our university with the biggest local medical school and one of the top medical schools in the region and all across world [5]. After successful completion of the training program, physicians who join any of the residency programs at our institution are offered a master's degree in higher specialty in medicine in addition to a residency training certificate which qualifies them to set for the Jordanian board exam.

Joining a postgraduate residency program and selecting the most suitable physicians to be admitted to a particular program require a considerable time and effort by the applicants and the selection committees. At the same time, anticipating success during residency and correlation with the admission selection criteria remains unclear [6]. There are no uniformly accepted criteria for the selection of the most suitable candidates. Although objective criteria might sound fairer, subjective evaluation tools are required to judge the non-cognitive competencies of the applicants [7].

At a national level, the total number of postgraduate training positions that are filled annually is less than 500. This means that only 20% of the 2019 graduates have the chance of being accepted at one of the residency programs in the country. The process of application and acceptance has never been reported in any of the Jordanian centers that offer this training. Therefore, we decided to review the current selection process conducted at our center and utilize this as a single-center model to serve a guide for resident selection. We are reporting the whole process of application, selection criteria, and the match results.

## 2 Methods

The purpose of this study is to propose a model for single-center resident selection for postgraduate residency programs based on a review of the selection criteria and match results at our institution in the year 2020. We conducted a cross-sectional study of the candidates who applied for residency programs at an independent center in Jordan in the year 2020. At our institution, there are a total of 96 positions distributed over 19 residency programs. Clinical training occurs at the university-affiliated hospital which is a 620-bed tertiary center staffed by more than two hundred academic consultants.

The eligibility criteria to apply for any of those programs include successful completion of a bachelor degree in medicine from any of the Jordanian medical schools or from a non-Jordanian school endorsed by the Ministry of higher education, successful completion of a one-year internship, possession of an unrestricted license from the Ministry of health, and an active registration within the Jordanian Medical Association.

Yearly in spring, the faculty of medicine and faculty of graduate studies at Jordan University of Science and Technology (JUST) announce the start of the application process. This is an electronic procedure where applicants who meet the eligibility requirements are given a thirty-day period to submit an application and upload all the necessary documents. All applicants are required to provide original or notarized documents to verify their medical school diploma, medical transcripts, internship completion, and unrestricted license to practice medicine in the country. Applicants are given the choice to rank up to four residency program specialties in the order of their interest. Applications that don't meet the eligibility criteria or not accompanied by the necessary documents are automatically rejected by the system. No application is considered after the deadline for the submission is reached. Thereafter, all the uploaded documents are reviewed and verified by the administrative members in the faculty of medicine and the faculty of graduate studies. Subsequently, all applicants are invited to set for a comprehensive structured medical exam that is held through a computerized system within the institution. The exam constitutes of 100 multiple-choice questions and all examinees get their scores released immediately once they submit their answers at the end of the exam. All applicants who don't achieve a minimum of 50% in the entry exam are excluded from the competition process.

After the exam, the applicants are arranged in a list according to a total score out of 100 distributed as follows: 25 points given to their cumulative GPA at graduation, 15 points to the medical school they graduated from, and 60 points to their performance in the online exam as determined by their score. These criteria were set by the deans’ council in order to provide very clear, transparent and objectively measured criteria about the selection process. Simultaneously, the total number of residency vacancies and their distribution among the nineteen programs are announced to all applicants at the beginning of the application process. Based on their order in the final list, a computerized system matches the applicants with their requested specialties. The applicants are called after that to sign a contract and to complete their registration process.

Collected data include gender, age, high-school score, medical school origin, medical school GPA, interval since graduation, entry exam score, and collective score out of 100 for each of the applicants. Data analysis was performed using excel and SPSS version 23 (SPSS Inc, Chicago, IL). Categorical variables were reported as numbers and percentages. Matching with either of the first two requested specialties was compared against the independent variables of selection criteria using chi-square distribution. To calculate the adjusted effect of the independent variables on the match results, a logistic regression was applied. Adjusted odds ratios (AOR) with 95% confidence interval (C.I) were reported as appropriate.

Administrative approval was obtained from the faculty of graduate studies at JUST to carry out this report. This work is reported based on STROCSS 2019 guidelines [8]. It has been registered at Research Registry database with unique identification number 5949. Participants’ data privacy and confidentiality are maintained as this study was conducted in compliance with the ethical standards per Helsinki declaration.

## 3 Results

A total of 785 eligible applicants submitted an electronic application to fill 96 positions at nineteen residency programs. Among the applicants, 56% were males, 686 graduated from Jordanian medical schools representing 87% of the applicants. 401 graduated from JUST medical school. Almost two thirds (512/785) were fresh graduates within the preceding year. Nearly half the number of applicants failed the entry exam and were excluded from the competition process. Of the 96 who matched, 50 (52%) were males, 84 (87.5%) were fresh graduates, 86 (90%) had more than 80% in their medical degree GPA, and 43 (45%) had a score of 70% or more in the entry exam. All 96 graduated from Jordanian medical schools and the majority (92/96) were from JUST medical school. Table 1.

**Table 1:** Applicant characteristics and credentials.

		Total		Matched	
		N = 785	Percent	N = 96	Percent
Gender	Male	443	56.4	50	52.1
	Female	342	43.6	46	47.9
					
Interval years	1	512	65.2	84	87.5
	2–3	182	23.2	12	12.5
	>3	91	11.6	0	0
					
High school score/100	98–100	248	31.6	57	59.3
	95–97.9	328	41.8	32	33.3
	90–94.9	129	16.4	6	6.3
	<90	80	10.2	1	1.1
					
MD score/100	>80	287	36.5	86	89.5
	60–79.9	477	60.8	10	10.5
	<60	21	2.7	0	0
					
MD origin	Jordan	686	87.4	96	100
	Non-Jordanian	99	12.6	0	0
					
MD school	JUST	401	51.1	92	95.8
	Other Jordanian	285	36.3	0	0
	Non-Jordanian	99	12.6	0	0
					
Exam score/100	>70	59	7.5	43	44.8
	50–69.9	343	43.7	53	55.2
	Fail	383	48.8	0	0
					
Total score/100	90–99	2	0.3	2	2
	80–89.9	24	3.1	23	24
	70–79.9	139	17.7	71	74
	60–69.9	223	28.4	0	0
	50–59.9	209	26.6	0	0
	Fail	188	23.9	0	0

MD: Medical degree.

JUST: Jordan University of Science and Technology.

Among the first two choices requested by the applicants, internal medicine (210) and general surgery (181) were the most requested specialties whereas emergency medicine (31), laboratory microbiology (14), and forensic medicine (12) programs were the least. The most competitive programs that were filled by the top 4% of the applicants were ophthalmology, otolaryngology and dermatology. Seventy-two out of 96 (75%) matched with either of their first two requested specialties. Table 2, Table 3.

**Table 2:** Distribution of the first two choices by specialty.

	Requested	Matched (n = 72)	Matched (%)
Anesthesia	47	3	6.4
Dermatology	81	2	2.5
Otolaryngology	96	3	3.1
Emergency medicine	31	0	0
Forensic medicine	12	1	8.3
Family practice	101	6	5.9
Internal medicine	210	13	6.2
Microbiology	14	1	7.1
Neurology	76	3	3.9
Neurosurgery	48	1	2.1
Obstetrics	125	7	5.6
Ophthalmology	109	3	2.8
Orthopedic surgery	95	4	4.2
Pathology	45	4	2.2
Pediatrics	107	7	6.5
Psychiatry	24	1	4.2
Radiology	90	5	5.5
General surgery	181	6	3.3
Urology	77	2	2.6

**Table 3:** Match results per request rank.

	N = 96	Percent
First choice	43	44.8
Second choice	29	30.2
Third choice	13	13.5
Fourth choice	11	11.5

The factors that increased the likelihood of matching with the first two choices include a graduation from JUST medical school with a GPA of more than 80% and an entry exam score of more than 70%. In a logistic regression model, the main factor associated with matching with the first two requested specialties was an entry exam score of more than 70% (AOR 8.7, 95% C.I. 2.4, 31.9). Table 4.

**Table 4:** Applicant characteristics and credentials according to match results.

		First two choices n = 72		3rd or 4th choices n = 24		*P value*
		Number	Percent	Number	Percent	
Gender	Male	37	51.4	13	54.2	0.81
	Female	35	48.6	11	45.8	
						
Interval years	1	62	86.1	22	91.7	0.72
	2–3	10	13.9	2	8.3	
	More than 3	0	0	0	0	
						
High school score/100	98–100	44	61.1	13	54.2	0.72
	95–97.9	26	36.1	6	25	
	90–94.9	2	2.8	4	16.7	
	Less than 90	0	0	1	4.1	
						
MD score/100	>80	66	91.6	20	84.3	0.24
	60–79.9	6	8.4	4	16.7	
	Less than 60	0	0	0	0	
						
MD school	JUST	70	97.2	22	91.7	0.55
	Other Jordanian	2	2.8	2	8.3	
	Non-Jordanian	0	0	0	0	
						
Exam score/100	>70^a^	40	55.6	3	12.5	0.0002
	50–69.9	32	44.4	21	87.5	
	Fail	0	0	0	0	
						
Total score/100	90–99	2	2.8	0	0	0.02
	80–89.9^b^	21	29.2	2	8.3	
	70–79.9	49	51	22	91.7	
	60–69.9	0	0	0	0	
	50–59.9	0	0	0	0	
	Fail	0	0	0	0	

MD: Medical degree.

JUST: Jordan University of Science and Technology.

a AOR 8.7, 95% C.I. (2.4, 31.9).

b AOR 5.1, 95% C.I. (1.1, 23.8).

## 4 Discussion

This report reviewed the 2020 model of the selection process of postgraduate residents to fill 96 positions at nineteen postgraduate residency programs at JUST, a major academic institution in Jordan. Globally, there are marked variations in the selection process of candidates for postgraduate training in different parts of the world. Some centers rely on locally established institutional criteria while others might have a national system utilized by all institutions [9].

The selection criteria followed by the residency programs to discern the most suitable candidates are divided into two big categories. The subjective criteria which study the non-cognitive skills of the candidates including communication skills, ethical approaches, and problem solving abilities. On the other hand, the objective criteria that depend on their measured scores during the medical school or at the entry exam reflect the candidates’ medical knowledge and clinical performance in a way that is less liable to selection bias [7].

In our institution, the majority of applicants are local graduates. Given the relatively small society and to avoid any selection bias in choosing the most suitable candidates for certain programs, we strictly implemented objectively measured criteria in our selection model. For this reason, personal interviews and recommendation letters, which are utilized by some other institutions worldwide, were not among the criteria used in our selection model [10]. With the limited number of positions, our report emphasized the importance of the entry exam as a tool to measure the applicants’ competency in medical knowledge in order to increase their chances to be accepted into a program of interest.

In the United States, the process of residency program match is controlled by the Association of the American Medical colleges (AAMC) through the National Resident Match Program (NRMP) which occurs at a national level every year using the internet-based Electronic Residency Application Service (ERAS) which has been implemented for more than two decades [11]. Among the NRMP selection criteria are the applicant's clinical performance during medical school years, United State Medical Licensing Exam (USMLE) scores, research experience, and the applicant's performance during the personal interview [11]. Some US studies have concluded that there is a positive correlation between the medical knowledge assessment tool utilizing the USMLE exam score as a marker and the performance in the in-training service exams [12]. On the other hand, some other reports showed that medical school performance didn't correlate with the resident performance in certain residency programs [6]. Some major centers in the UK include the applicant's research experience and the reputation of their publication journals among the essential criteria for their acceptance into a residency program [13]. This is not the case in most US centers during the resident selection but is definitely included during recruitment for clinical fellows after graduating from residency programs.

In our region in the Middle East, there is a paucity of literature about the selection criteria utilized by postgraduate training centers. In Kuwait, Marwan et al. studied the perception of the program members about the criteria affecting the selection for residency programs [14]. In their survey, the authors identified the personal interview, clinical performance, and medical knowledge reflected by GPA and honors during medical study as the most important criteria in resident selection [14].

In our model, the main factors that determine the candidates’ likelihood of acceptance into his/her requested specialty are the cumulative GPA at graduation from medical school and the achieved score during the structured entry exam. As a matter of fact, the score of the entry exam was found to be the most significant determinant. Unfortunately, there are no reports about the selection criteria implemented by other centers in Jordan to compare our approach with. Still, there is a consensus among most Jordanian residency program centers that passing an entry exam is an essential prerequisite for the admission process.

Personal interviews have been reported as an essential component by most authors who studied the selection process among different residency specialties in the US. Given the large number of applicants for the US residency programs including international medical graduates, interviews are expected to clarify some of the vague points in the applications, help to judge the clinical performance and medical knowledge, and to assess the communication skills and problem solving abilities of the applicants. In our system, since most of our applicants graduated from the same medical school, they are well known to most of the selection committee members and their clinical background is well documented in their diplomas and transcripts. Simultaneously, in a relatively small community, the use of such a subjective measure may be subject to selection bias that interferes with transparency of the selection process.

Our report highlighted that certain specialties are highly demanded by our applicants compared with others. In our institution, it was clear that ophthalmology, otolaryngology and dermatology were very competitive. This was evident by the fact that all available positions were filled by the top 4% of the applicants. The most-in-demand medical specialties vary by institutions and countries. In the US, the most demanded specialties nowadays are family practice, emergency medicine, and psychiatry. Factors that determine the demand of certain specialties over others might be related to experience of the students during their clinical rotations and most likely to the availability of post-graduation job opportunities and the feasibility of further sub-specialization [15].

The main limitation in our report is the application of identical criteria to all programs within the institution which restrict the chance for some applicants to join their most favorite programs if they don't achieve high cumulative score. Also, the lack of reports from other Jordanian centers and the extreme paucity of reports from the region preclude comparison with other centers from similar settings.

In conclusion, the selection process for postgraduate residency programs at our institution is highly competitive. To avoid selection bias, transparent and objectively measured criteria are applied in the selection model. Clinical performance and medical knowledge reflected by the cumulative GPA and entry exam score are the most significant determinants for acceptance. Regardless of the criteria implemented by different programs, it remains unclear if the selection criteria are indicative of the quality of specialists after completion of their training [16,17]. Therefore, follow up studies to correlate the applicant attributes with future success are needed to establish more helpful guidelines for a more standardized selection.

## Funding

None.

## Provenance and peer review

Not commissioned, externally peer reviewed.

## Ethical approval

An administrative approval was obtained from the faculty of graduate studies at Jordan University of Science and Technology.

## Author contribution

**WK:** Made substantial contribution to study design and literature review. Participated in data collection, analysis and interpretation. Involved in drafting the manuscript and revising it critically for important intellectual content. **NO:** Participated in data analysis and interpretation. Involved in revising the manuscript critically for important intellectual content. **BA:** Participated in data acquisition and interpretation. Involved in drafting the manuscript and revising it critically for important intellectual content. **KE:** Made substantial contribution to study design. Participated in drafting the manuscript and revising it critically for important intellectual content.

## Registration of research studies

1. Name of the registry: Research registry.

2.Unique Identifying number or registration ID: 5949.

3. Hyperlink to your specific registration (must be publicly accessible and will be checked): https://www.researchregistry.com/browse-the-registry#home/registrationdetails/5f47318174d2dd00159c8c0a/

## Guarantor

All authors give final approval for the version to be published and agreed to be accountable for all aspects of the work.

## Consent

Consent was waived.

## Declaration of competing interest

All authors have no conflict of interest.

## References

[1] “Tawjihi - Wikipedia https://en.wikipedia.org/wiki/Tawjihi#cite_note-1.

[2] “Ministry of Higher Education & Scientific Research http://www.mohe.gov.jo/en/Pages/default.aspx/FAQs.aspx.

[3] Faleh Tamimi A. (2010). Medical education in Jordan Medical education in Jordan. Med. Teach..

[4] Jordan Medical Council “ https://www.jmc.gov.jo/.

[5] Jordan University of Science and Technology “ http://www.just.edu.jo/Pages/Default.aspx.

[6] Stohl H.E., Hueppchen N.A., Bienstock J.L. (Sep. 2010). Can medical school performance predict residency performance? Resident selection and predictors of successful performance in obstetrics and gynecology. J. Grad. Med. Educ..

[7] Lemay J.F., Lockyer J.M., Collin V.T., Brownell A.K.W. (Jun. 2007). Assessment of non-cognitive traits through the admissions multiple mini-interview. Med. Educ..

[8] Agha R., Abdall-Razak A., Crossley E., Dowlut N., Iosifidis C., Mathew G. (Dec. 2019). STROCSS 2019 Guideline: strengthening the reporting of cohort studies in surgery. Int. J. Surg..

[9] Batalden P., Leach D., Swing S., Dreyfus H., Dreyfus S. (Aug. 2002). General competencies and accreditation in graduate medical education. Health Aff..

[10] Stohl H.E., Hueppchen N.A., Bienstock J.L. (Sep. 2011). The utility of letters of recommendation in predicting resident success: can the ACGME competencies help?. J. Grad. Med. Educ..

[11] Green M., Jones P., Thomas J.X. (2009). Selection criteria for residency: results of a national program directors survey. Acad. Med..

[12] Thundiyil J.G., Modica R.F., Silvestri S., Papa L. (Jan. 2010). Do United States medical licensing examination (USMLE) scores predict in-training test performance for emergency medicine residents?. J. Emerg. Med..

[13] Sinha S., Sinha A., Sinha S., Bhan C., McConnachie A., Knowles C.H. (Mar. 2010). Selection matters-A regional survey of UK consultant opinion on selection into postgraduate surgical and medical training. J. Surg. Educ..

[14] Marwan Y., Ayed A. (2013). Selection criteria of residents for residency programs in Kuwait. BMC Med. Educ..

[15] (2020). 10 Most In-Demand Medical Specialties.

[16] Dore K.L. (2010). The reliability and acceptability of the multiple mini-interview as a selection instrument for postgraduate admissions. Acad. Med..

[17] Jerant A. (Mar. 2019). Do admissions multiple mini-interview and traditional interview scores predict subsequent academic performance? A study of five California medical schools. Acad. Med..

